# Supplementary material for: Novel computed tomography-based tools reliably quantify plant reproductive investment
Source: J Exp Bot. 2017 Dec 23;69(3):525–35. doi: 10.1093/jxb/erx405 (PMC5853293; doi:10.1093/jxb/erx405)
Supplement: Appendix S1 [file erx405_suppl_appendix_s1.pdf]

Appendix S1. Loss of contrast of developing seeds in selected European orchid species and comparison with *Arabidopsis thaliana*

In the main text, we describe new methods to count ovules (and pollen grains) from orchids via Computed Tomography (CT) by making use of the preferential enrichment of the contrasting agent phosphotungstic acid (PTA) in protein-rich tissues (Staedler *et al*., 2013). These new methods allow the first direct count of the extremely numerous ovules of Orchidoideae (see main text). During natural fertilization, it is possible that only part of these extremely numerous ovules develop to seeds. Can the methods we present in the main text also allow us to measure the fertilization rates of the ovules within orchid fruits? Because the contrasting agent phosphotungstic acid bind primarily to protein (Hayat, 2000), and therefore most strongly to undifferentiated/meristematic tissues, we expect that staining intensity will decrease in developing orchid fruits, and that counting is not possible. In order to assess if our observations can be generalized to other taxa, we also compared our results with anthetic flowers and developing fruits of *Arabidopsis thaliana*.

Orchid flowers from four species (see table S1) from the bottom of inflorescences were hand pollinated with entire pollinia from different individuals of the same population. After two weeks, developing fruits were harvested and fixed as in the main text. Infiltration with phosphotungstic acid lasted for 6 weeks. Mounting was as in the main text with the difference that developing fruits were mounted into 500µl pipette tips (Brand, Carl Roth GmbH+Co KG). Scanning equipment is as in main text. Scanning conditions are summarized in Table S2.

For all four Orchidoideae species studied, in all anthetic gynoecia scanned it was possible to threshold the ovules, *i.e.***,** to set a greyscale threshold value so that the remainder voxels overwhelmingly belong to the ovules (Fig. S2a-b, only *Orchis militaris* shown). This, in turn makes it possible to count ovules based on their volumes (see main text). In early late fruits, however, the contrast of the developing has decreased, and they cannot be segregated from the background via greyscale thresholding anymore (Fig. S2c-d, only *Orchis militaris* shown). It is therefore not possible to count developing seeds of the studied species via greyscale thresholding of the CT scan data.
In *Arabidopsis thaliana*, in anthetic flowers it is possible to threshold the ovules (Fig. S2e-f). This would make it possible to count ovules based on volume. Later in development, four and six days after fertilization (Fig. S2g and Fig. S2i), it is not possible to threshold the developing seeds anymore (Fig. S2h and and Fig. S2j). Nine days after fertilization (Fig. S2k), however, it is possible to threshold the developing embryos (Fig. S2l).

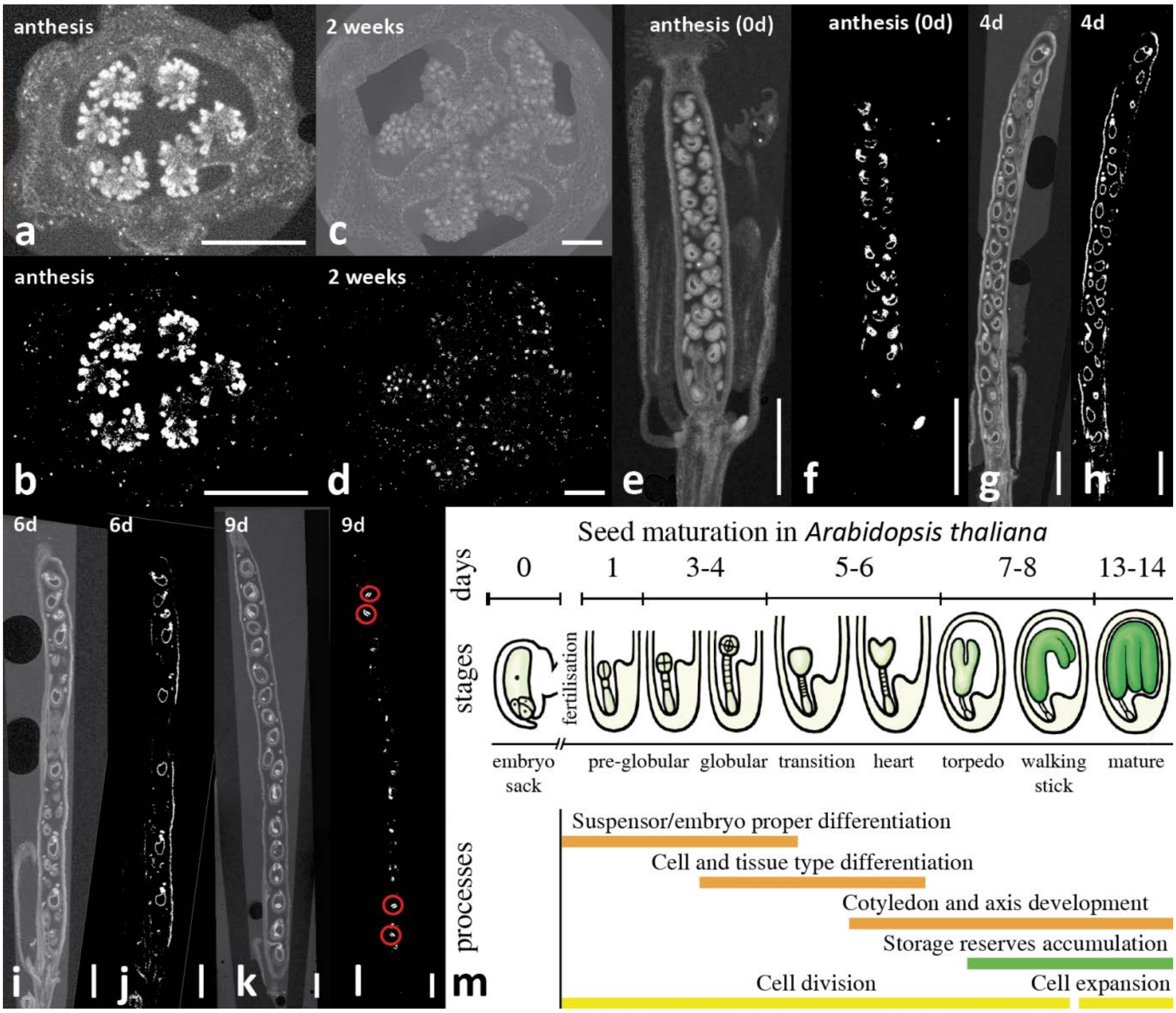

Fig. S2 Greyscale thresholding on flowers and young fruits of *Orchis militaris* and *Arabidopsis thaliana*. a-d, reconstructed transverse sections of flower and fruit of *Orchid militaris*. e-l, reconstructed longitudinal sections (parallel to the septum) of flower and fruit of *A. thaliana* pAP1::AP1-GR ap1-1 cal-5 mutant. Days on figure are days after anthesis. a, flower before thresholding. b, flower after thresholding (thresholding of the ovules possible). c, early late fruit (two weeks old) before thresholding. d, early late fruit fruit after thresholding (thresholding of the developing seeds not possible). e, anthetic flower. f, anthetic flower after thresholding (thresholding of the ovules possible). g, early fruit. f, early fruit after attempted thresholding (thresholding of the developing seeds possible). i, middle fruit. j, middle fruit after thresholding (thresholding of the developing seeds not possible). k, late fruit. l, late fruit after thresholding (thresholding of the developing embryos possible), in red circles embryos with both cotyledons visible. m, schematic representation of *Arabidopsis* seed development, modified from (Le *et al*., 2010). Seed cartoons adapted from (Mansfield and Bowman, 1993) and not drawn to scale. Fruits started opening after day nine. Scale bar = 500µm.

Counting ovules in anthetic flowers is possible both in the Orchidoideae species studied and in *A. thaliana*. This is possibly due to the fact that ovules contain cytoplasm-rich meristematic/undifferentiated tissues that, after fertilization, allow their maturation into seeds (Wobus and Weber, 1999). Counting ovules via CT, based on the selective enrichment of phosphotungstic acid (PTA) in protein-rich (cytoplasm-rich) tissues (meristematic/undifferentiated) is thus a technique that is possibly broadly applicable. Because counting ovules on 3D scan data also offers crucial advantages (it is not destructive and more straightforward than counting on sections or electron micrographs), we currently use scans to count ovules in other, unrelated taxonomic groups, such as: *Primula*, Primulaceae (Staedler *et al*., submitted) and Merianiae, Melastomataceae (Agnes Dellinger, personal communication).

Counting developing seeds in orchids may not be possible due to the specific properties of orchid seeds, themselves linked to the specificities of orchid life cycle. In nature, the seeds of orchids must parasitize fungi in order to develop into photosynthetic individuals (Arditti, 1992). In accordance with a parasitic juvenile stage, the seeds contain but few (lipid) storage reserves (Manning and Van, 1987) and are both extremely numerous and extremely small (Arditti, 1992). Within the seeds, the mature embryos themselves are very small, and generally occupy only a very small part of the volume inside the seed coat, leaving a large amount of air space (Arditti and Ghani, 2013; Yam *et al*., 2002). Mature orchid embryos commonly contain *ca*. 30 to 200 cells and are usually between 80 and 150 µm wide (Yam *et al*., 2002). The very small orchid embryos may thus not constitute tissues large enough to be thresholded and safely identified as such (*i.e.*, differentiate them from small bright artefacts).

Counting developing seeds in *Arabidopsis* is less straightforward than counting ovules. During seed development in *Arabidopsis*, the outer tissues of the ovule, the integuments differentiate into the seed coat and die (Haughn and Chaudhury, 2005), which would lead to a loss of capacity to bind PTA and thus a loss of contrast; this would explains why integuments cannot be thresholded in developing seeds (see Fig. S2f *vs.* Fig. S2h, j, l). Concomitantly, the embryo and endosperm develop (see Fig. S2m): until shortly before maturity, the embryo is undergoing intensive cell division (see Fig. S2m, processes).

At this point in development, the embryo is capable of binding much more PTA than the surrounding tissues, and can thus be thresholded. Compared to that of orchids, the mature *Arabidopsis* embryos are fairly large: they contains thousands of cells and are *ca.* 500µm wide (Bassel *et al*., 2014). This volume allows them to be safely distinguished from artefacts. The ideal time frame to use CT to count seeds in *Arabidopsis* is thus during the late seed development stages (such as in Fig. S2k-l), but possibly not the very last stages. During the very last stages of seed development in *Arabidopsis*, cell divisions cease and give way to cell expansion and storage reserve accumulation (see Fig. S2m), which is expected to lead to contrast decrease because the reserve storage is largely in the form of lipids (Mansfield and Briarty, 1992), to which PTA does not bind (Hayat, 2000).

Developing seeds, unlike ovules may or may not contain enough of the tissues that allow for the selective contrast increase with PTA on which counting relies. The presence of significantly large meristematic tissues such as a present in a sufficiently large developing embryo is critical for the successful use of PTA to count developing seeds. In order to estimate fertilization rates in plants with very numerous seeds, approaches that make use of contrasting agents that bind to reserve storage molecules, e.g., lipid-binding contrasting agents such as OsO_4 ((Hayat, 2000) but see (Staedler *et al*., 2013) limitations) and starch-binding contrasting agents such as Lugol's iodine (Hayat, 2000) could be promising to assess fertilization rates in orchids and other taxa. Therefore, although CT is a robust and non-destructive tool to measure reproductive investment, its use to assess fertilization rates has to be considered on a case by case basis.

References

Arditti J. 1992. *Fundamentals of orchid biology*. New York: John Wiley & Sons.

Arditti J, Ghani A. 2013. Numerical and physical properties of orchid seeds and their biological implications (vol 145, pg 367, 2000). *New Phytologist* **200**.

Bassel GW, Stamm P, Mosca G, de Reuille PB, Gibbs DJ, Winter R, Janka A, Holdsworth MJ, Smith RS. 2014. Mechanical constraints imposed by 3D cellular geometry and arrangement

modulate growth patterns in the Arabidopsis embryo. *Proceedings of the National Academy of Sciences* **111**, 8685-8690.
Haughn G, Chaudhury A. 2005. Genetic analysis of seed coat development in Arabidopsis. *Trends in Plant Science* **10**, 472-477.
Hayat M. 2000. *Principles and techniques of electron microscopy*. Cambridge: Cambridge University Press.
Le BH, Cheng C, Bui AQ, Wagmaister JA, Henry KF, Pelletier J, Kwong L, Belmonte M, Kirkbride R, Horvath S. 2010. Global analysis of gene activity during Arabidopsis seed development and identification of seed-specific transcription factors. *Proceedings of the National Academy of Sciences* **107**, 8063-8070.
Manning J, Van SJ. 1987. The Development and Mobilisation of Seed Reserves in Some African Orchids. *Australian Journal of Botany* **35**, 343-353.
Mansfield S, Bowman J. 1993. Embryogenesis. *Arabidopsis: An Atlas of Morphology and Development, J. Bowman, ed (Berlin: Springer-Verlag)*, 349-362.
Mansfield S, Briarty L. 1992. Cotyledon cell development in Arabidopsis thaliana during reserve deposition. *Canadian Journal of Botany* **70**, 151-164.
Staedler YM, Bielowski WR, Chartier M, Manafzadeh S, Keller B, Yang B, Sun H, Conti E, Schönenberger J. submitted. Reproductive investment and floral polymorphism: an attempt to shed a new light on homostylous Primula.
Staedler YM, Masson D, Schönenberger J. 2013. Plant tissues in 3D via X-ray tomography: simple contrasting methods allow high resolution imaging. *PLoS One* **8**, e75295.
Wobus U, Weber H. 1999. Seed maturation: genetic programmes and control signals. *Current Opinion in Plant Biology* **2**, 33-38.
Yam TW, Yeung EC, Ye X-L, Zee S-Y, Arditti J. 2002. Embryology-Seeds. In: Kull T, Arditti J, eds. *Orchid Biology: Reviews and Perspectives, VIII*. Dordrecht: Springer Netherlands, 287-385.
