# Supplementary material for: Novel computed tomography-based tools reliably quantify plant reproductive investment
Source: J Exp Bot. 2017 Dec 23;69(3):525–35. doi: 10.1093/jxb/erx405 (PMC5853293; doi:10.1093/jxb/erx405)
Supplement: Supplementary Data S1 [file erx405_suppl_supplementary_data_s1.pdf]

Supplementary data S1. Choice of ANOVA test

We did not perform parametric tests because our data is nonparametric. Variables tested: number of ovules (n° ovules), number of pollen grains (n° pollen), and pollen to ovule ratio (P/O).

Shapiro-Wilk normality test ($p<0.05$ implies that data is not normally distributed):

n° ovules:	W = 0.85194	p-value = 3.277e-07
n° pollen:	W = 0.78335	p-value = 7.737e-08
P/O:	W = 0.81865	p-value = 5.745e-07

Bartlett test of homogeneity of variances ($p<0.05$ implies that the variance is not homogeneous):

n° ovules /species:	Bartlett's K-squared = 48.57	df = 7	p-value = 2.755e-08
n° pollen /species:	Bartlett's K-squared = 84.247	df = 7	p-value = 1.869e-15
P/O/species:	Bartlett's K-squared = 15.006	df = 7	p-value = 0.03592]

We could log transform the variable "n° ovules" and "P/O" to meet the parametric criteria, but not the variable "n° pollen". We thus decided to use nonparametric tests for the three variables.
