# Supplementary material for: Novel computed tomography-based tools reliably quantify plant reproductive investment
Source: J Exp Bot. 2017 Dec 23;69(3):525–35. doi: 10.1093/jxb/erx405 (PMC5853293; doi:10.1093/jxb/erx405)
Supplement: Supplementary Figure S1 [file erx405_suppl_supplementary_figure_s1.pdf]

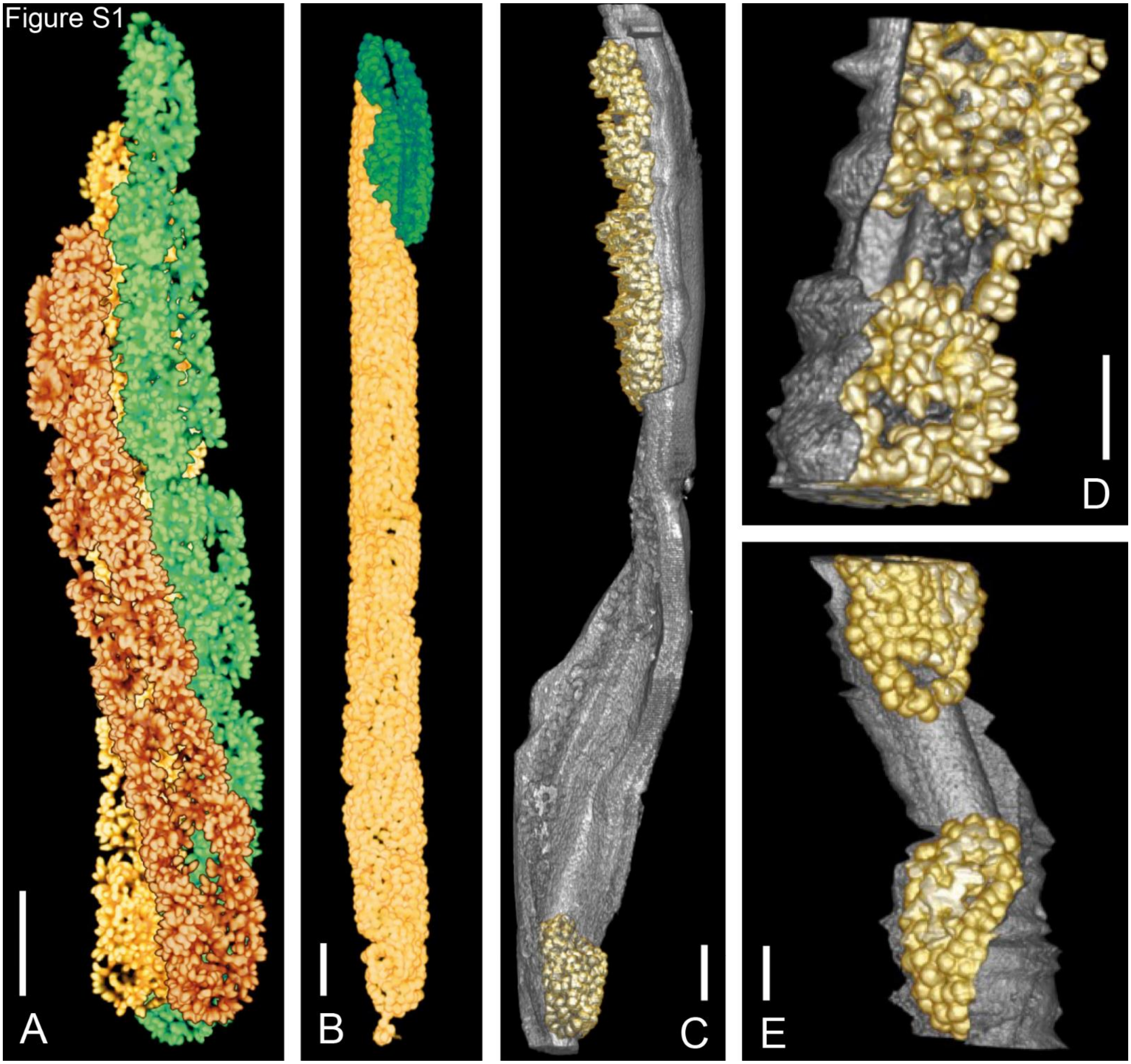

Fig. S1. Lack of homogeneity in ovule distribution. Ovule distribution differences between flowers from the bottom of the inflorescence and flowers from the top of the inflorescence. Ovules from different placentae are in different colours; placentae are in grey; distal end of gynoecia towards the top of the page. (A) Ovules from gynoecium of bottom flower of *Dactylorhiza incarnata*. (B) Top flower of *D. majalis*, one placenta is completely missing, and one is present only for less than 25% of the length of the ovary (in green).
(C) Flower from top of inflorescence of *D. majalis*; more than 50% of the placenta length is devoid of ovules.
(D) Flower from top of inflorescence in *Anacamptis pyramidalis*, small gap of ca. 5% of placenta length.
(E) Flower from top of inflorescence in *D. incarnata*, small gap of ca. 14% of placenta length. Scale bars = 500 µm.
